# Editorial: Recent advances in papillary thyroid carcinoma: Lymph node metastasis

**DOI:** 10.3389/fendo.2022.1084034

**Published:** 2022-11-24

**Authors:** Jose Federico Carrillo, Erivelto Martinho Volpi

**Affiliations:** ^1^ Head and Neck Department, National Institute of Cancerology (INCAN), Mexico City, Mexico; ^2^ Head and Neck Department, Oncology Center, Oswaldo Cruz German Hospital, São Paulo, São Paulo, Brazil

**Keywords:** lymph node metastasis, papillary thyroid carcinoma, contrast enhanced evaluation in thyroid cancer, thermal ablation in thyroid cancer lymph node metastasis, distant related metastasis to lymph node metastasis

Papillary thyroid carcinoma (PTC) has been associated at initial presentation with a high rate of lymph node metastases(LNM). Until recently, this factor was not considered significant regarding oncologic outcomes However, at present time, many reports consider LNM as an established risk factor associated to Disease free and overall survival.

In this topic, Zhang et al. address the presence of lateral lymph node metastases (LLNM) in microcarcinoma and find tumor size ≥5 mm, multifocality, upper lobe location and presence of central lymph node metastases (CLNM) associated significantly in multivariate analyses. This fact underscores the possibility of a more integrated treatment in these small lesions, with better long term results.

In similar way Chang et al. construct a nomogram with a wider spectrum of PTC cases, where they include plasma thyroglobulin (PS-Tg) level as well as male, age, maximal tumor diameter, multifocality, and Extrathyroid extension (ETE) to predict CLNM, and another one including same factors and the presence of CLNM to detect LNM.

Both aforementioned manuscripts confirm findings reported by others (1, 2), and the present tendency to perform in a more efficient way a comprehensive treatment (including central and or lateral neck dissection during initial surgery), with improvement in the final oncologic results and quality of life of patients.

Aside, Fang et al. underline the role of contrast enhanced Ultrasound (CEUS) as a novel tool to increase the preoperative diagnostic accuracy of this method, with hyper-enhancement, centripetal perfusion, and ring enhancement as characteristics of malignancy, which could avoid unnecessary Fine needle aspiration biopsy (FNAB) in selected cases, specifically in median to high risk malignancies, to proceed in a swifter and more straightforward surgical strategy.

Another manuscript by Lai et al. describes a computer-based method to predict LNM in an effort, as well, to design better surgical strategies, along with prediction of patients with high risk of LNM: they find random forest analyses as the more accurate method to define risk in PTC, and consequently of LNM. A web-based tool is supplied,and represents a potential better definition of differentiated thyroid cancer risk- although with lack of external validation- and a pioneer effort regarding this issue in urgent need of improvement at present.

Although with a significant number of patients with low-intermediate risk factors, the need to proceed with a bilateral central neck dissection specifically regarding the posterior lymph nodes to the right recurrent laryngeal nerve (LN-prRLN), is reported by Zhou et al. They identify ETE, tumor size ≥1.5cm and anterior to recurrent nerve LNM(LN-arRLN), associated to posterior LN-prRLN, and as an indication to proceed with bilateral central lymph nodes dissection (CLND) to avoid recurrences, and potentially improve recurrence-free survival (RFS).

Non-surgical therapies like ultrasound guided thermal ablation-including radiofrequency, laser, and microwave techniques to treat LN recurrences of the neck have been described in a novel systematic review analyses by Zhang et al. These techniques have also been described related to primary benign and malignant thyroid nodules (3), and in a very similary way -aside from the small numbers of patients and the very limited world regions where these procedures are performed- the results are promising in terms of biochemical and structural follow-up. A major complication rate of 12%, and 70% mean reduction in size of the recurrent lesions are deterrants to wide application of this technique, which is a feasible and promising tool for very selected patients who refuse surgery or have major comorbidities.

The issue of metastatic disease (DM) in thyroid carcinoma in relation to lymph node metastatic disease has been raised scarcely, and a recent paper by Ho (4) provides an assessment of this matter, where the lateral lymph node burden, -lymph nodes number (LNN) and lymph node ratio (LNR)-was related to DM-either lung metastases(LM) or bone metastes(BM)-only in classic PTC and tall cell(TC variants).

In an extensive retrospective analyses, Wang et al. not only confirm the aforementioned findings but discriminate according to age the predictive value of LNN and LNR regarding presence of DM. LN burden was related to LM in younger patients(≤55 years), and to LM as well as BM in older patients (≥55years). Lack of impact of N1a disease regarding DM is also suggested which supports a more conservative approach in management of T1a cases.

The reports in this issue not only underline the role of LNM regarding differentiated thyroid cancer, but the need to discriminate from the start point of therapy which patients are candidates for more extensive treatments. In same manner new approaches and surgical techniques are needed to treat recurrent neck disease because of the high risk of the operative approaches existent (in previously treated with surgery and radioiodine fields), - as already mentioned in Zhang et al. report- with one of these being the wire guided needle approach for surgical recurrences in thyroid carcinoma (5, 6).

## Author contributions

EV. Design, writing, draft, and editing of manuscripts related to the topic. JC Design, writing, draft, and editing of manuscripts related to the topic. All authors contributed to the article and approved the submitted version.

## Conflict of interest

The authors declare that the research was conducted in the absence of any commercial or financial relationships that could be construed as a potential conflict of interest.

## Publisher’s note

All claims expressed in this article are solely those of the authors and do not necessarily represent those of their affiliated organizations, or those of the publisher, the editors and the reviewers. Any product that may be evaluated in this article, or claim that may be made by its manufacturer, is not guaranteed or endorsed by the publisher.
